# Senescent T-Cells Promote Bone Loss in Rheumatoid Arthritis

**DOI:** 10.3389/fimmu.2018.00095

**Published:** 2018-02-01

**Authors:** Johannes Fessler, Rusmir Husic, Verena Schwetz, Elisabeth Lerchbaum, Felix Aberer, Patrizia Fasching, Anja Ficjan, Barbara Obermayer-Pietsch, Christina Duftner, Winfried Graninger, Martin Helmut Stradner, Christian Dejaco

**Affiliations:** ^1^Department of Rheumatology and Immunology, Medical University of Graz, Graz, Austria; ^2^Department of Endocrinology and Diabetology, Medical University of Graz, Graz, Austria; ^3^Department of Internal Medicine VI, Innsbruck Medical University, Innsbruck, Austria; ^4^Rheumatology Service, South Tyrolean Health Trust, Hospital Bruneck, Bruneck, Italy

**Keywords:** T-lymphocyte, rheumatoid arthritis, osteoporosis, aging, IL-15

## Abstract

**Objective:**

T-cells are critical players in the pathogenesis of osteoporosis in patients with rheumatoid arthritis (RA). Premature senescence of lymphocytes including the accumulation of senescent CD4^+^ T-cells is a hallmark feature of RA. Whether T-cell senescence is associated with bone loss in RA patients is elusive so far.

**Methods:**

This includes a prospective study of consecutive patients with RA (*n* = 107), patients with primary osteopenia/-porosis (*n* = 75), and healthy individuals (*n* = 38). Bone mineral density (BMD) was determined by dual-energy X-ray absorptiometry scan. Flow cytometry, magnetic-associated cell sorting, and cell culture experiments were performed to analyze the pro-osteoclastic phenotype and the function of senescent CD4^+^CD28^−^ T-cells.

**Results:**

Patients with osteopenia/-porosis yielded a higher prevalence of senescent CD4^+^CD28^−^ T-cells than individuals with normal BMD, in the RA, as well as in the non-RA cohort. Receptor activator of nuclear factor kappa-B ligand (RANKL) was expressed at higher levels on CD4^+^CD28^−^ T-cells as compared to CD28^+^ T-cells. Stimulation with interleukin-15 led to an up-regulation of RANKL expression, particularly on CD28^−^ T-cells. CD4^+^CD28^−^ T-cells induced osteoclastogenesis more efficiently than CD28^+^ T-cells.

**Conclusion:**

Our data indicate that senescent T-cells promote osteoclastogenesis more efficiently than conventional CD28^+^ T-cells, which might contribute to the pathogenesis of systemic bone loss in RA and primary osteoporosis.

## Introduction

Bone loss is one of the most common comorbidities of patients with rheumatoid arthritis (RA). Depending on the population studied, 10–56% of RA patients suffer from osteoporosis, and consequently RA patients are at an increased risk of low-trauma fractures as compared to the general population (1, 2).

In healthy individuals, bone homeostasis is maintained by a balance between bone formation and bone resorption (3). A link between inflammation and bone loss has been suggested for decades, and it was supported by *in vitro* observations and animal models showing enhanced bone resorption under the influence of pro-inflammatory cytokines including interleukin (IL)-1, IL-6, and TNF-α (4). T-cells are one of the most important promoters of osteoclastogenesis, and the first evidence for the capacity of T-cells to cause bone loss was provided by Kong et al. in 1999 by illustrating that T-cell-produced receptor activator of nuclear factor kappa-B ligand (RANKL) triggered osteoclastogenesis directly in a mouse model of adjuvant-induced arthritis (5). More recently, another study showed that T-cell-deficient mice were resistant to bone loss using a mouse model of postmenopausal osteoporosis (6). Subsequently, numerous other studies have investigated the potential role of T-cells to interfere with bone homeostasis (7, 8).

Premature immunosenescence including the accumulation of senescent CD4^+^ T-cells seems to be a hallmark feature of RA (9, 10). Senescent T-cells are characterized by the loss of CD28, eroded telomeres, the lower content of T-cell receptor excision circles, the expression of pro-inflammatory molecules, and the gain of effector functions (11–13). Notably, senescent CD28^−^ T-cell prevalence correlated with disease severity in RA (9, 14).

The role of immunosenescence in the context of osteoporosis, however, is elusive so far. The aim of this study was to investigate whether senescent CD4^+^28^-^ T-cells are associated with early bone loss in RA patients.

## Materials and Methods

### Study Population

This was a prospective study on 107 consecutive patients with RA meeting the 2010 ACR/EULAR criteria (15) and 113 consecutive individuals without RA (non-RA) referred for dual-energy X-ray absorptiometry (DXA) scan. These non-RA subjects were subsequently classified either “healthy” or having “primary osteoporosis/osteopenia” according to the WHO criteria (osteoporosis in case of *T*-score of less or equal to −2.5 and osteopenia if *T*-score was between −2.5 and −1.0) (16). Patients were recruited at the outpatient clinics of the Rheumatology and Endocrinology Departments of the Medical University Graz, respectively. Detailed family and medical history including disease duration, prior and current treatments, as well as fracture risk assessment tool (FRAX) (17) data identifying clinical risk factors for osteoporosis and osteoporotic fractures were obtained from each individual. Clinical visits of RA patients were performed at baseline and subsequently for every 6 months up to 2 years. Non-RA controls underwent a baseline visit only. Synovial fluid samples were obtained from RA patients undergoing routine joint aspiration.

This study was approved by the Institutional Review Board of the Medical University Graz, and written informed consent was obtained from each individual.

### Bone Mineral Density

Measurement of BMD by DXA was performed according to routine protocols. All RA patients underwent the scans at baseline, after 1 year and after 2 years, whereas controls underwent baseline scans only. BMD was assessed at the lumbar spine (L1–L4) and the left (or the right, if not possible otherwise) hip and femoral neck. Vertebral bodies with significant degenerative changes or compressive fractures (as judged by the investigating physician) were excluded from analysis, and the mean BMD was calculated using the remaining vertebral bodies (applicable only in case of at least two adjacent analyzable vertebrae). BMD was expressed in g/cm^2^ and has a *T*-score, which is derived from the SD of bone-healthy comparators.

### Laboratory Parameters and Parameters of Bone Metabolism

Laboratory parameters at baseline and every 6 months during follow-up were determined (detailed in Figure S1 in Supplementary Material).

### Peripheral Blood Mononuclear Cells (PBMCs) and Cell Culture

Peripheral venous blood or synovial fluid was drawn from each individual, and PBMCs were isolated by Histopaque density-gradient centrifugation. The total cell number was determined by a Beckmann Coulter. Cells were cultured at 1 × 10^6^ cells/ml in RPMI 1640 containing 10% fetal calf serum, 2 mM l-glutamine, 100 U/ml penicillin, and 100 μg/ml streptomycin in the presence of 20 U/ml human-recombinant IL-2 (SIGMA, Vienna, Austria) and initial stimulation with 10 μg/ml plate-bound anti-CD3 Ab (eBioscience).

For stimulation assays, PBMCs were stimulated with 100 ng/ml of IL-15, IL-6, and, TNF-α (all Sigma-Aldrich) for 3 days or with 10 μg/ml plate-bound anti-CD3 Ab overnight. Unstimulated cells were served as a negative control.

### Flow Cytometry

Surface and intracellular staining of freshly isolated PBMCs was performed according to routine protocols and using appropriate combinations of antibodies for the detection of CD3, CD4, CD8, CD28, CD45RA, CD45RO, CD57 (all Becton Dickinson, San Diego, CA, USA), and RANKL (eBioscience). For intracellular staining of cytokines and intracellular RANKL, Golgi transport was inhibited by Brefeldin A (10 ng/ml) and Monensin (10 ng/ml) for 4 h prior to intracellular staining. Appropriate isotype controls were used. Stained cells were measured using a FACS Canto II (Becton Dickinson), and data analysis was conducted with DIVA software and FlowJo.

### Isolation of T-Cell Subsets

For functional assays, CD4^+^ T-cells were isolated by the positive selection of PBMCs labeled with magnetic-bead-conjugated anti-human CD4 mAbs using MACS MultiSort Kit and autoMACSPro according to manufacturer’s instructions (Miltenyi Biotech, Amsterdam, The Netherlands). Purified CD4^+^ T-cells were then separated into the CD28^+^ and CD28^−^ fractions by another sorting step using FACS technology (FACS Aria). For validation, flow cytometry was performed to determine the purity (>95%) of selected cells.

### Isolation of Monocytes and Osteoclast Differentiation

Separation of the monocyte fraction from PBMCs was carried out by the plastic adherence technique using 96-well plates (0.3 × 10^6^ cells/well) and serum-free α-MEM supplemented with 1% penicillin/streptomycin, 10 ng/ml M-CSF, and 1 ng/ml TGF-β (Peprotech, Hamburg, Germany). Monocytes were obtained from healthy donors. After 5–7 days, monocyte-enriched cells were washed with PBS and treated with trypsin/EDTA for detachment. Monocytes of 5 × 10^4^ were then cultivated for 13 days in the absence or presence of 1 × 10^5^ CD4^+^CD28^+^ and CD4^+^CD28^−^ T-cells from RA patients or 1 ng/ml RANKL (Peprotech) as a positive control. Medium was replaced twice weekly. Visual inspection confirmed the continued adherence of cells with each change of medium.

Osteoclast differentiation was evaluated by staining cells for tartrate-resistant acid phosphatase (TRAP) using the Leukocyte Acid Phosphatase Kit (Sigma-Aldrich, Vienna, Austria) according to the manufacturer’s instructions. Differentiated osteoclasts were defined as TRAP positive and multinucleated cells with ≥3 nuclei.

### Statistical Analysis

All statistical analyses were performed using the SPSS program, version 23 (Chicago, IL, USA). The Kolmogorov–Smirnov test was used to analyze the distribution of the variables. In case of a parametric distribution of continuous variables, data were reported as mean and SD, and we applied the two-sided Student’s *t*-test (comparison of two groups) for comparisons. In case of a non-parametric distribution, the results were described as median and range, and we conducted the Mann–Whitney *U*- and the Kruskal–Wallis tests. Paired data were compared with the Wilcoxon test. Correlation between variables was evaluated by Spearman’s rank correlation coefficient.

## Results

### Patients’ Characteristics

In total, 220 (107 RA and 113 non-RA) patients were included in the investigation. Clinical baseline characteristics are depicted in Table 1. FRAX data, full laboratory parameters, and results of genetic testing for a predisposition of lactose intolerance are shown in Tables S1–S3 in Supplementary Material. In the RA cohort, 12 (12.5%), 39 (40.6), and 26 (27.1) out of the 96 patients with available SDAI values had high-, moderate-, and low-disease activity, respectively, and 19 (19.8) patients were in remission (18). Six (6%) RA patients had early disease (≤2 years of duration). Clinical characteristics of RA patients at follow-up visits are shown in Table S4 in Supplementary Material.

**Table 1:** Patients’ characteristics.

	Non-RA	RA	*p*-Value
Number	113	107	
Age (years)^a^	61.3 (±10.5)	62.6 (±11.5)	0.372
Female, *n* (%)	96 (85)	81 (75.7)	0.148
Disease duration (years)^b^	n.a.	12.3 (0–46)	
Bone mineral density	*n* = 111	*n* = 105	
Normal, *n* (%)	38 (34.2)	28 (26.7)	0.303
Osteopenia, *n* (%)	31 (27.9)	55 (52.4)	<0.001
Osteoporosis, *n* (%)	44 (39.6)	22 (21)	0.005
DAS			
SDAI^b^	n.d.	12.1 (0–50.7)	
DAS28^b^	n.d.	3.3 (0.3–7.1)	
Laboratory data			
ESR (mm/1st h)^b^	n.d.	15 (1–66)	
CRP (mg/l)^b^	n.d.	3.5 (0–52)	
Current medication			
Corticosteroids, *n* (%)	1 (0.9)^c^	25 (23.4)	
Biologicals, *n* (%)			
Anti-TNFα	0	27 (25.2)	
Tocilizumab	0	6 (5.6)	
Abatacept	0	13 (12.1)	
Rituximab	0	3 (2.8)	
DMARDs, *n* (%)			
Methotraxate	0	59 (55.1)	
Leflunomide	0	16 (15)	
Sulfasalazine	0	6 (5.5)	
Other	0	5 (4.7)	
NSAIDs, *n* (%)			
Regularly	0	13 (12.1)	
On demand	0	74 (69.2)	
Osteoporosis treatment, n (%); n in normal/osteopenia/osteoporosis			
Bisphosphonates	29 (25.7) 2/10/17	18 (16.8) 2/8/8	0.102
Vitamin D	47 (41.6) 9/14/24	33 (30.8) 6/16/11	0.048
Calcium	43 (38.1) 2/13/28	47 (43.9) 8/24/15	0.490
Raloxifene	2 (1.8) 0/1/1	0	
Strontium ranelate	2 (1.8) 0/0/2	0	
Denosumab	1 (0.9) 0/0/1	0	

*^a^Mean (±SD)*.

*^b^Median (range)*.

*^c^Due to asthma*.

*CRP, C-reactive protein (0–5 mg/l); DAS28, Disease Activity Score 28; DMARD, disease-modifying anti-rheumatic drugs; ESR, erythrocyte sedimentation rate (0–30 mm/h); *n*, number; n.a., not applicable; n.d., not determined; NSAID, non-steroidal anti-inflammatory drugs; RA, rheumatoid arthritis; sDAI, simplified disease activity index*.

### No Changes of BMD in RA Patients Over Time

Only minor changes of BMD could be observed in RA patients for 2 years in this study (for details, see Table 2).

**Table 2:** BMD of RA patients over time of clinical visits.

	Baseline (*n* = 96)	12 months (*n* = 60)	24 months (*n* = 54)
BMD spine^a^ (gm/cm^2^)	1.047 (±0.173)	1.074 (±0.153)	1.084 (±0.168)
BMD hip^a^ (gm/cm^2^)	0.913 (±0.156)	0.923 (±0.128)	0.911 (±0.123)
BMD femoral neck^a^ (gm/cm^2^)	0.880 (±0.144)	0.883 (±0.122)	0.874 (±0.116)

*^a^Data are depicted as mean (±SD)*.

A progression of bone loss as indicated by a loss of 5% of BMD over time (24 months) was observed in 17 (15.9%) (lumbar spine 13/total hip 4/femoral neck 6) RA patients. These 17 patients did not significantly differ from patients with stable BMD concerning medication and FRAX scores and lymphocyte subsets (data not shown). Baseline disease activity parameters correlated weakly with progressive bone loss at the lumbar spine (corr_coeff_ with DAS28 = 0.319, *p* = 0.033; corr_coeff_ with SDAI = 0.319, *p* = 0.029). In addition, a correlation of baseline osteocalcin levels with osteoporosis progression of the femoral neck (corr_coeff_ = 0.410, *p* = 0.002) was observed.

### Senescent T-Cells Accumulate in Patients with Reduced Bone Mass

Rheumatoid arthritis is associated with premature senescence of T-lymphocytes (11, 13), and the accumulation of senescent CD4^+^CD28^−^ T-cells has been linked with disease severity (9, 14). The possible involvement of senescent T-cells in RA-associated and primary bone loss, however, has not been studied so far.

**Figure 1:**
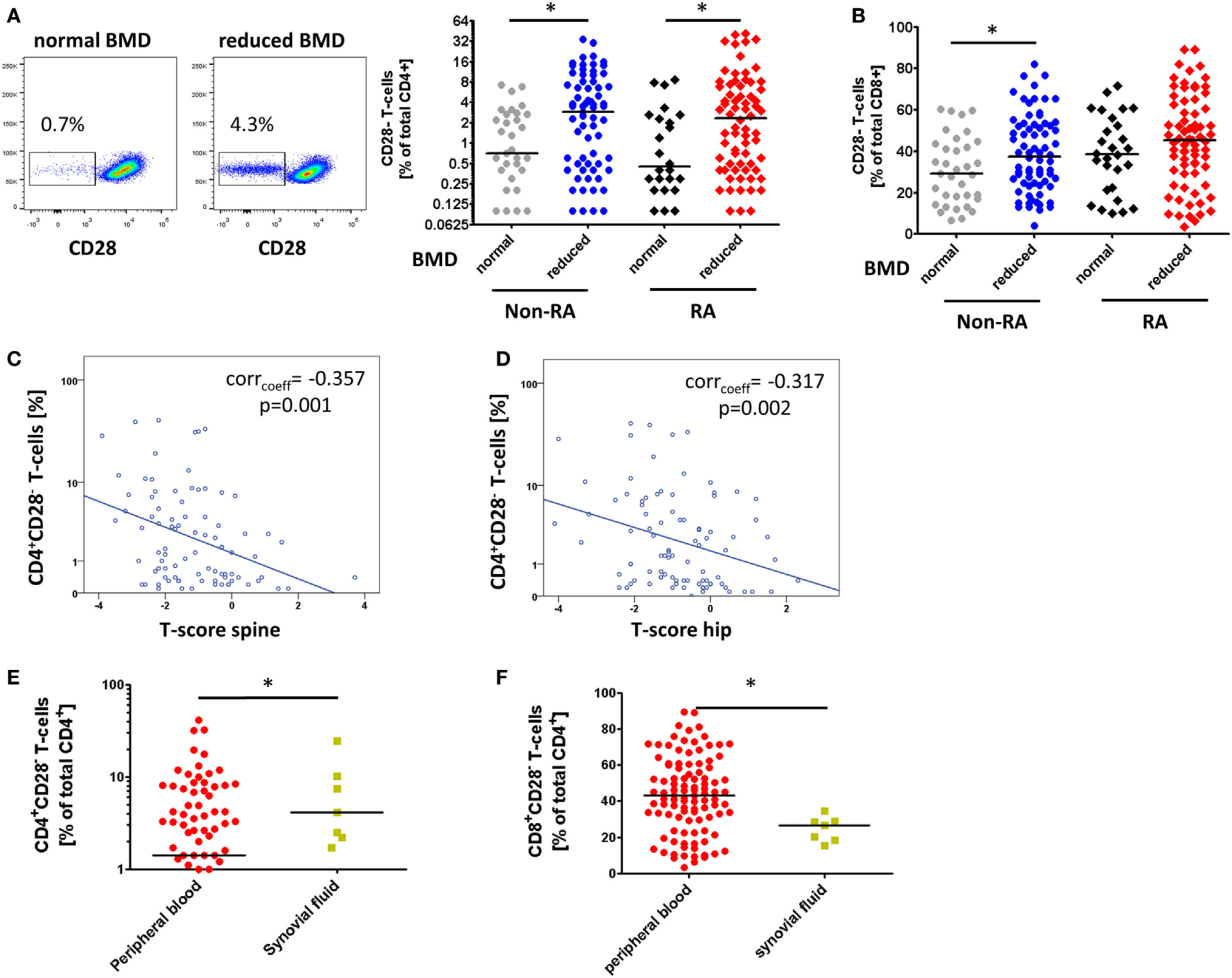
The accumulation of CD4^+^CD28^−^ T-cells in patients with reduced bone mineral density (BMD). Graphs show **(A)** representative dot plots of freshly isolated CD4^+^CD28^−^ T-cells in rheumatoid arthritis (RA) patients with normal and reduced BMD and CD4^+^CD28^−^ T-cell frequencies in RA and non-RA cohort; **(B)** frequencies of freshly isolated CD8^+^CD28^−^ T-cells in healthy individuals and patients with primary osteopenia/-porosis; **(C)** correlation of CD4^+^CD28^−^ T-cells of RA patients with *T*-score of the lumbar spine and **(D)** the hip; **(E)** frequencies of freshly isolated CD4^+^CD28^−^ T-cells; and **(F)** frequencies of CD8^+^CD28^−^ T-cells in synovial fluid of RA patients (yellow, *n* = 7) compared to peripheral blood (red). *p ≤ 0.05, **(A,B,E,F)** Mann–Whitney *U*-test, **(C,D)** Spearman’s rank correlation.

Rheumatoid arthritis patients with reduced bone mass showed elevated frequencies of CD4^+^CD28^−^ T-cells compared to patients with normal bone mass [2.2% (0–41.2) vs. 0.5% (0.1–8.6), *p* = 0.013, Figure 1A], whereas the prevalence of CD8^+^CD28^−^ T-cells was not different between the groups [45.2% (3.4–89.3) vs. 37.4% (10–61), *p* = 0.197, Figure 1B]. RA patients with osteoporosis showed the highest levels of senescent CD4^+^ T-cells [4.4% (0.2–39.7)] as compared to RA patients with osteopenia [1.4% (0–41.2)] and patients with normal bone mass [0.5% (0.1–8.6), Figure S1A in Supplementary Material].

In the non-RA cohort, we also noted increased frequencies of CD4^+^CD28^−^ T-cells [2.9% (0.1–34) vs. 0.7% (0.1–7.3), *p* = 0.010, Figure 1A] as well as CD8^+^CD28^−^ T-cells [38.3% (4.1–82.1) vs. 28.7% (6.6–59.8), *p* = 0.035, Figure 1B] in patients with reduced bone mass compared to healthy controls. Patients with osteopenia showed the highest levels of CD4^+^CD28^−^ T-cells [3.7% (0.1–34)] followed by patients with osteoporosis [2.4% (0.1–19.2)] and healthy controls [0.7% (0.1–7.3), Figure S1B in Supplementary Material].

In the RA cohort, frequencies of senescent CD4^+^CD28^−^ and CD8^+^CD28^−^ T-cells correlated significantly with BMD of the lumbar spine (CD4: corr_coeff_ = −0.361, *p* = 0.001, Figure 1C; CD8: corr_coeff_ = −0.248, *p* = 0.016), the hip (CD4: corr_coeff_ = −0.310, *p* = 0.003, Figure 1D; CD8: corr_coeff_ = −0.204, *p* = 0.045), and the femoral neck (CD4: corr_coeff_ = −0.275, *p* = 0.009; CD8: corr_coeff_ = −0.175, *p* = 0.088). Such correlations, however, were not observed in the non-RA group.

During follow-up, the frequencies of T-cell subsets remained stable in the RA cohort. None of the T-cell subsets at baseline predicted any change of BMD over time (data not shown).

To investigate the prevalence of CD4^+^CD28^−^ T-cells at the site of inflammation, we analyzed the samples of RA synovial fluid. The frequency of CD4^+^CD28^−^ T-cells was elevated compared to peripheral blood [4.1% (1.7–24.3) vs. 1.4% (0–41.2), *p* = 0.047, Figure 1E]. By contrast, the prevalence of CD8^+^CD28^−^ T-cells was diminished [26.6% (15.4–34.5) vs. 43.1% (3.4–89.3), *p* = 0.013, Figure 1F].

### Elevated Surface RANKL Expression by Senescent T-Cells

We next investigated whether senescent T-cells express RANKL, which is a key mediator of bone loss (19).

**Figure 2:**
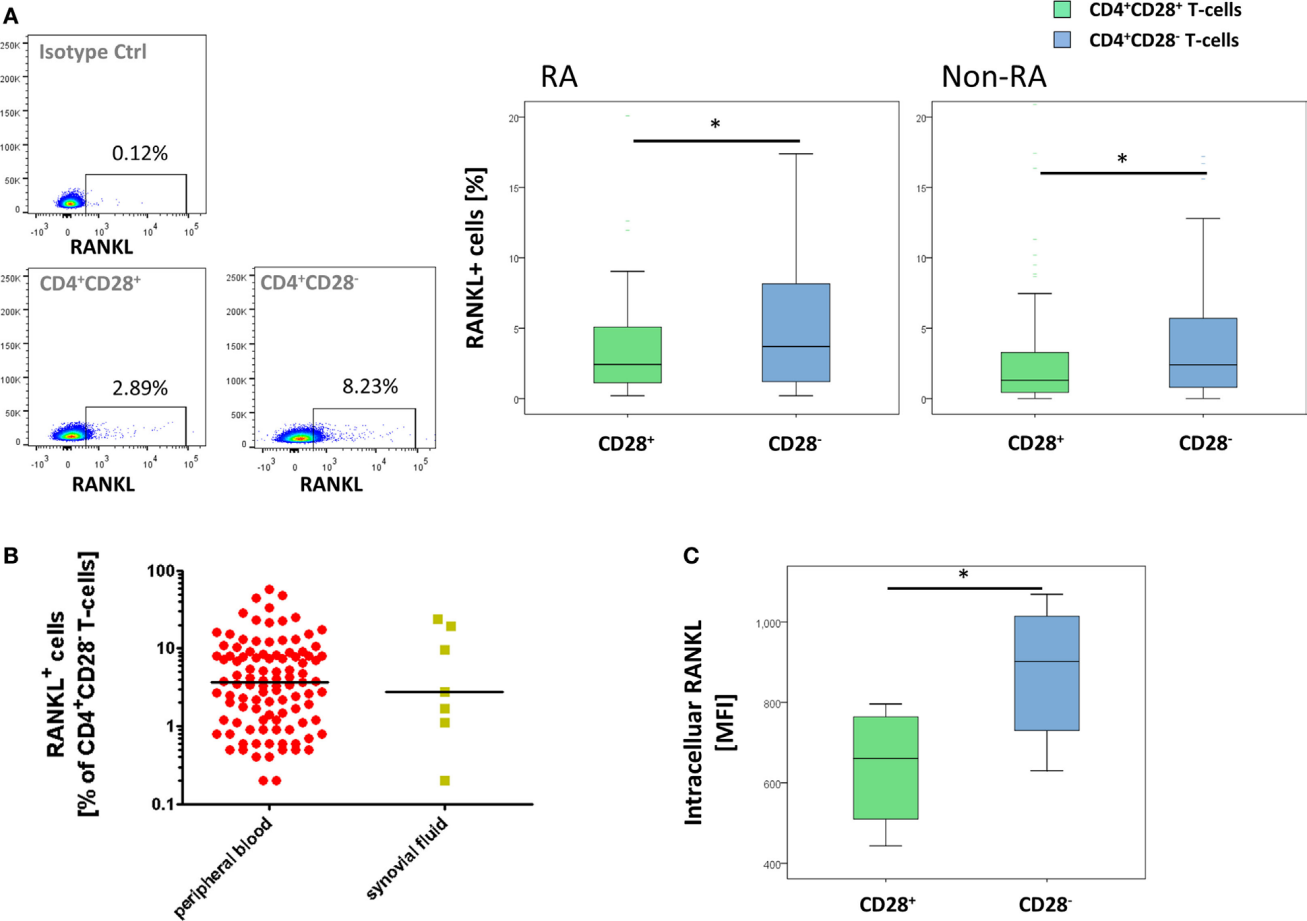
Increased receptor activator of nuclear factor kappa-B ligand (RANKL) expression by CD4^+^CD28^−^ T-cells. Graphs show **(A)** representative dot plots of freshly isolated RANKL^+^ cells as well as isotype control staining in CD4^+^CD28^+^ T-cells and frequencies of RANKL^+^ cells out of CD4^+^CD28^−^ (blue) and CD4^+^CD28^+^ (green) T-cells in RA (*n* = 107) and non-RA cohort (*n* = 113); **(B)** prevalences of freshly isolated RANKL^+^CD4^+^CD28^−^ T-cells in synovial fluid (yellow) compared to peripheral blood (red) and **(C)** a representative histogram as well as median fluorescence intensity (MFI) of intracellular RANKL in freshly isolated CD4^+^CD28^+^ T-cells (green) and senescent CD4^+^ T-cells (blue) of RA patients (*n* = 6). For all experiments, cells were analyzed directly *ex vivo*. *p ≤ 0.05 **(A–C)** Mann–Whitney *U*-test.

In the RA cohort, surface RANKL was significantly higher in CD4^+^CD28^−^ T-cells compared to CD28^+^ T-cells [3.7% (0.2–57.9) vs. 2.4% (0.2–35.4), *p* = 0.001, Figure 2A]. Within the CD28^+^ T-cell subset, memory CD45RO^+^ T-cells produced more surface RANKL than naïve CD45RA^+^CD4^+^ T-cells [2.7% (0.2–38) vs. 2.2% (0.2–30.5), *p* < 0.001, Figure S2A in Supplementary Material]. In CD4^+^CD28^−^ T-cells isolated from synovial fluid, RANKL expression was comparable to that of CD4^+^CD28^−^ T-cells isolated from peripheral blood [3.7% (0.2–57.9) vs. 2.8% (0.2–24.1), *p* = 0.893; Figure 2B]. Intracellular production of RANKL was higher in CD4^+^CD28^−^ T-cells compared to that in CD4^+^CD28^+^ T-cells [MFI: 901.5 (630–1069) vs. 660.5 (443–796), *p* = 0.028, Figure 2C] in patients with RA. Within the CD4^+^CD28^+^ population, memory CD45RO^+^ T-cells produced more intracellular RANKL than naïve CD45RA^+^ T-cells [781.5 (544–880) vs. 589.5 (373–669), *p* = 0.027, Figure S2C in Supplementary Material].

Results in the non-RA cohort were similar: surface RANKL production was significantly higher in CD4^+^CD28^−^ T-cells compared to that in CD28^+^ T-cells [2.4% (0–32.1) vs. 1.3 (0–34.8), *p* = 0.023, Figure 2A]. Again, memory CD4^+^ T-cells produced more surface RANKL than naïve CD4^+^ T-cells [1.4% (0–30.8) vs. 1.2 (0–45.3), *p* < 0.001, Figure S2B in Supplementary Material].

Each T-cell subset (i.e., naïve, memory, and senescent) in the RA cohort expressed more surface RANKL compared to the respective subset in the non-RA group (naïve: *p* < 0.001, memory: *p* = 0.001, senescent: *p* = 0.017).

### IL-15 Promotes Upregulation of Surface RANKL Expression

TNF-α, IL-6, and IL-15 are cytokines that are strongly associated with the pathogenesis of RA and were also reported to promote osteoclastogenesis (20–22). We therefore tested the ability of these cytokines to alter RANKL expression in different T-cell subsets.

**Figure 3:**
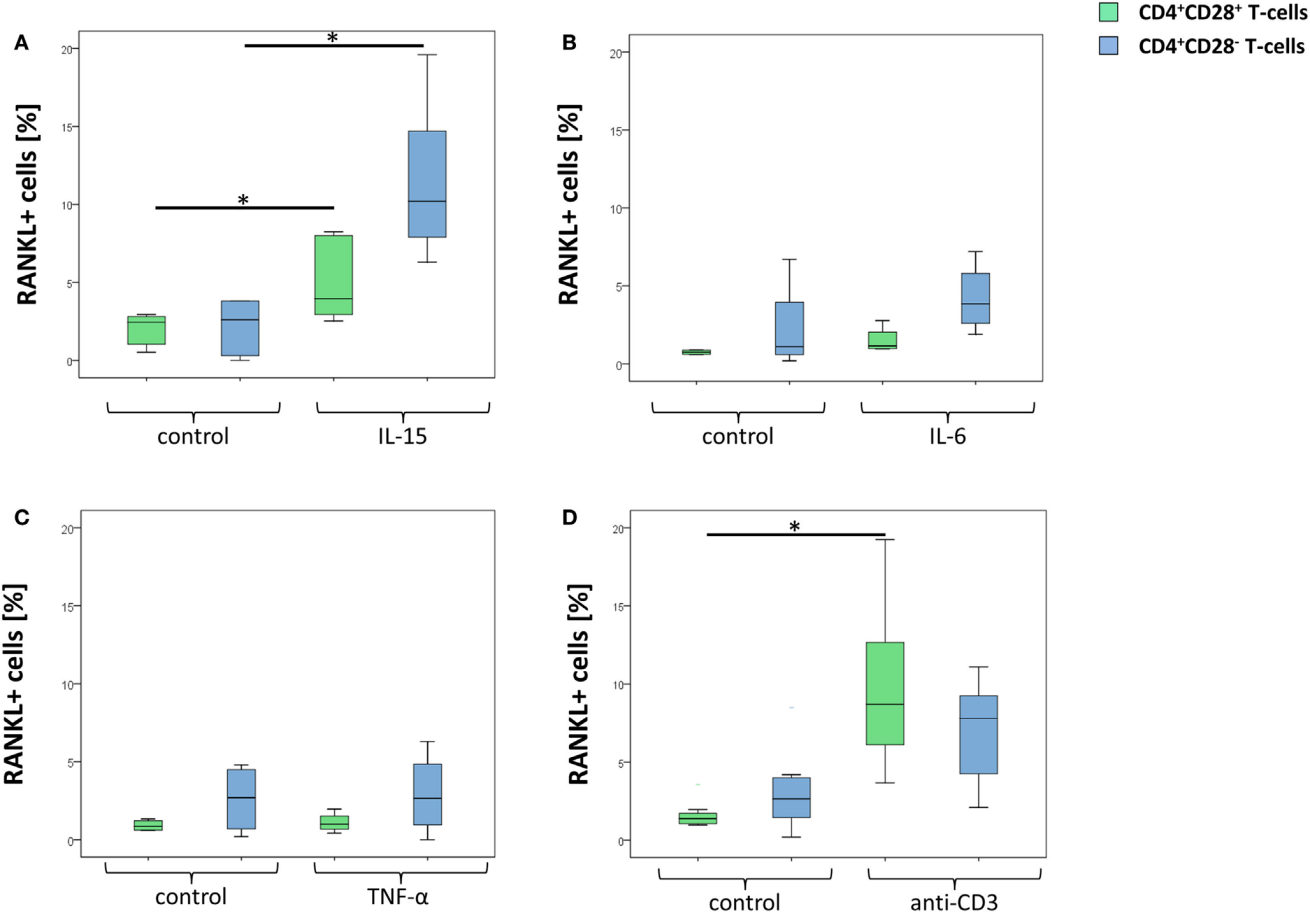
Increased receptor activator of nuclear factor kappa-B ligand (RANKL) expression following stimulation with IL-15. Graphs show **(A)** representative dot plots as well as prevalences of RANKL^+^ cells in CD4^+^CD28^+^ T-cells (green) and senescent CD4^+^ T-cells (blue) following stimulation with 100 ng/ml IL-15 (*n* = 5), **(B)** 100 ng/ml IL-6 (*n* = 5) and **(C)** 100 ng/ml TNF-α (*n* = 5) for 3 days or with **(D)** 10 μg/ml anti-CD3 antibody (*n* = 8) overnight. Cells were isolated from rheumatoid arthritis patients. *p ≤ 0.05 **(A–D)**, Wilcoxon test.

As depicted in Figure 3A, IL-15 significantly enhanced surface RANKL expression on both the CD28^+^ and the CD28^−^ CD4^+^ T-cell populations. The effect, however, was more prominent in CD28^−^ (5.4-fold higher expression compared to unstimulated cells) compared to CD28^+^ T-cells (3.5-fold). Within the CD28^+^ subset, IL-15 upregulated RANKL more strongly on memory (4.5-fold) compared to naïve T-cells (1.5-fold).

TNF-α as well as IL-6 failed to alter RANKL expression in all T-cell subsets (Figures 3B,C). Stimulation with anti-CD3 led to an increase of surface RANKL expression on CD28^+^ T-cells (7.9-fold), whereas senescent T-cells remained almost unaffected (Figure 3D).

Intracellular production of RANKL was modestly enhanced by anti-CD3 in CD28^+^ (1.5-fold) and CD28^−^ T-cells (1.3-fold), whereas stimulation with IL-15 did not further enhance RANKL production on either cell subset.

### Senescent T-Cells Effectively Induce Osteoclastogenesis

**Figure 4:**
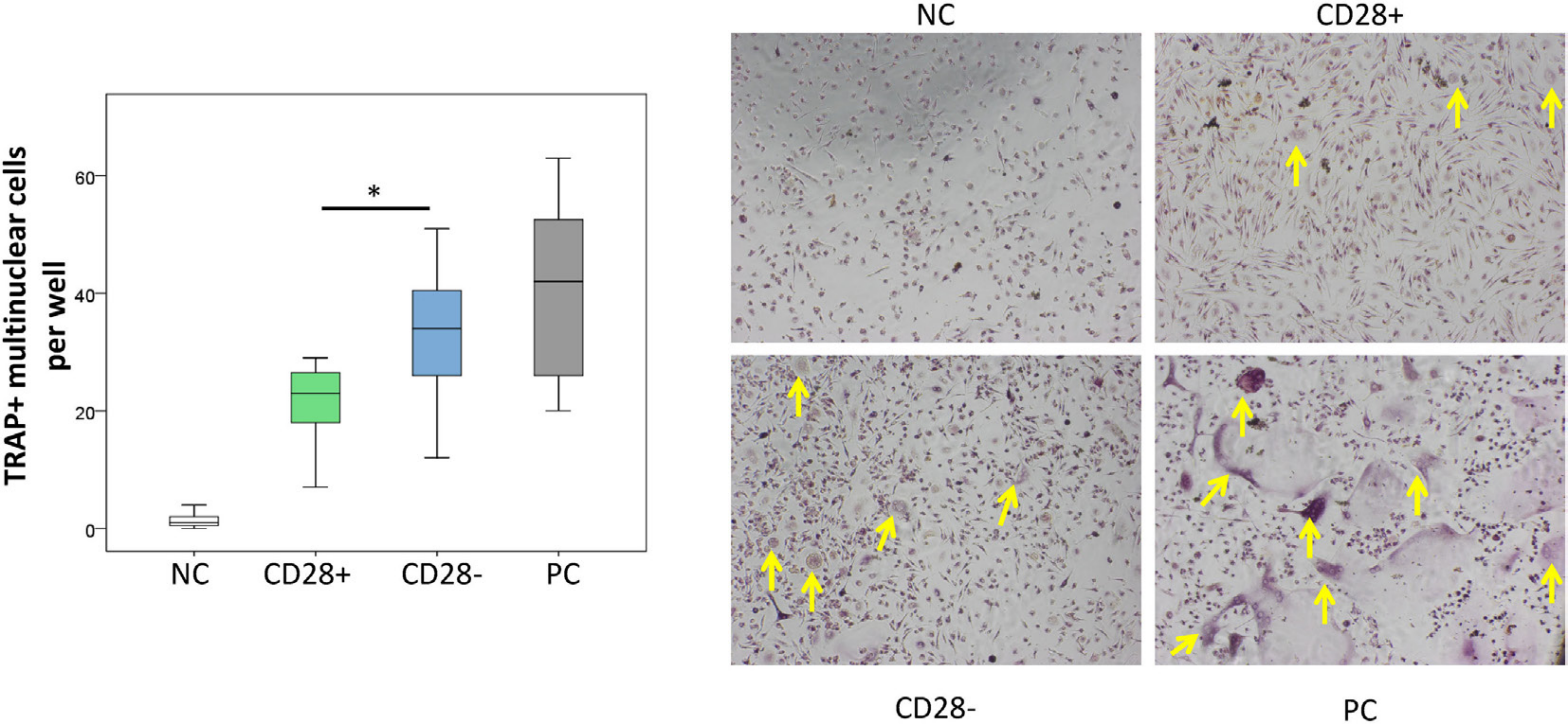
Enhanced osteoclastogenesis in the presence of CD4^+^CD28^−^ T-cells. The graph shows the number of tartrate-resistant acid phosphatase (TRAP^+^) multinuclear cells per well of monocytes in the absence of soluble receptor activator of nuclear factor kappa-B ligand (sRANKL) [negative control (NC), white], in the presence of CD4^+^CD28^+^ T-cells (green) or CD4^+^CD28^−^ T-cells (blue), and monocytes in the presence of sRANKL [positive control (PC), gray]. Representative images are given, and TRAP^+^ multinuclear cells are indicated with yellow arrows. All T-cell subsets were isolated from rheumatoid arthritis patients. *n* = 7, *p ≤ 0.05, Mann–Whitney *U*-test.

To evaluate whether the increased RANKL expression on CD28^−^ as compared to CD28^+^CD4^+^ T-cells results in a higher capacity to induce osteoclastogenesis *in vitro*, osteoclast differentiation assays were conducted. A higher number of TRAP + multinuclear cells and larger areas of bone resorbtion were observed in co-cultures of monocytes and senescent T-cells as compared to cultures involving CD28^+^ T-cells [34 osteoclasts/well (12–51) vs. 23 (7–29), *p* = 0.046, Figure 4 and Figure S3 in Supplementary Material]. Osteoclasts generated in the presence of either CD28^−^ or CD28^+^ T-cells were smaller and less intensively stained for TRAP than monocytes differentiated in cultures containing recombinant-soluble RANKL.

## Discussion

In the present work, we show that patients with systemic bone loss have a higher prevalence of circulating senescent CD4^+^CD28^−^ T-cells than individuals with normal BMD. RANKL is expressed at higher levels on senescent CD4^+^ T-cells compared to that on CD28^+^ T-cells, and its production can be stimulated with IL-15, a key cytokine in the pathogenesis of RA. Senescent CD4^+^ T-cells induce osteoclastogenesis more efficiently than CD28^+^ T-cells.

Several studies demonstrated that T-cells are involved in the bone-remodeling system and that RANKL-expressing T-cells promote local and systemic osteoporosis (5, 6). Besides, it has been demonstrated that senescent CD4^+^ T-cells were increased in patients with severe disease manifestations, and at the same time, these patients were at an increased risk of osteoporosis (23). These findings support our conclusion that senescent CD4^+^CD28^−^ T-cells play an important role in the promotion of osteoporosis in RA as well as in non-RA individuals. Interestingly, we observed similar frequencies of CD4^+^CD28^−^ T-cells in our RA and non-RA cohorts. Since our non-RA population contains an increased number of patients with reduced bone mass, this group is not comparable to healthy controls from other studies. Taken together, these findings lead to the conclusion that extra-articular and systemic manifestations are the main drivers of CD28^−^ T-cell emergence in RA and that bone loss and systemic inflammation might also increase the prevalence of these cell subsets in other populations (24, 25).

We observed that senescent CD4^+^ T-cells expressed higher levels of RANKL than CD28^+^ T-cells. In mouse models of aging, B-cells and bone marrow cells from aged mice also yielded higher RANKL levels as compared to young animals, suggesting that cells from the hematopoietic lineage gain RANKL expression along with cellular senescence (26, 27). Aged T-cells further acquire a senescence-associated secretory phenotype (“inflammaging”), leading to a pro-inflammatory milieu which favors bone loss (10, 28).

IL-15 is a key cytokine in the pathogenesis of RA. Elevated levels of IL-15 were found in RA synovium, and the concentrations of IL-15 in peripheral blood correlated strongly with disease activity (29, 30). Moreover, genetic variants in the IL-15 gene were reported to be associated with the progression of joint destruction in RA (31). In our study, IL-15 stimulation led to an increased RANKL expression in naïve, memory, and aged CD4^+^ as well as CD8^+^ T-cells. This up-regulation was most prominent in memory and aged compared to naïve lymphocytes. The pivotal role of IL-15 in promoting osteoclastogenesis is supported by a number of studies. Park et al., for example, reported that IL-15 up-regulates RANKL expression in rheumatoid synovial fibroblasts (32), and Okabe et al. showed, more recently, that IL-15 and RANKL act synergistically to induce osteoclastogenesis (33). Another study noted that mice lacking the IL-15 receptor have a higher BMD and a decreased number of osteoclasts. Interestingly, these mice showed an impairment of T-cell-dependent osteoclast activation and RANKL production (20).

Our study has some limitations: first, the capability of senescent CD4^+^ T-cells to induce osteoclastogenesis was not verified *in vivo*. Unfortunately, mouse models on CD4^+^CD28^−^ T-cells are not available so far, and therefore we have to rely on *in vitro* experiments as well as clinical studies to investigate the role of these cell subsets in rheumatic diseases. Nevertheless, we were able to show that these cells accumulate at sites of inflammation and retain a pro-osteoclastogenic phenotype. Second, we chose to include consecutive patients from our outpatients’ clinic, and therefore the patient cohort is heterogeneous with various treatments including corticosteroids and therapeutics for osteoporosis. Third, the progression of bone loss was observed only in a minority of RA patients, resulting in a lack of power to investigate whether the baseline prevalence of senescent T-cells would have been a predictor of the progression of bone loss. Furthermore, we did not observe an association between senescent T-cells and parameters of bone metabolism.

Taken together, our study establishes a link between senescent T-cells and bone loss in humans. CD4^+^CD28^−^ T-cells accumulate in patients with reduced BMD and exhibit a pro-osteoclastogenic phenotype *in vitro* which is further enhanced by IL-15. This cell population might thus contribute to the pathogenesis of RA-associated and primary bone loss.

## Ethics Statement

This study was approved by the Institutional Review Board of the Medical University Graz, and written informed consent was obtained from each individual.

## Author Contributions

JF, BO-P, WG, RH, VS, FA, EL, CDu, MS, and CDe designed the research study. JF, RH, PF, VS, EL, FA, and AF conducted the experiments and acquired data. JF, CDu, PF, MS, and CDe analyzed data. BO-P and WG provided reagents. JF, MS, and CDe wrote the manuscript.

## Conflict of Interest Statement

The authors declare that the research was conducted in the absence of any commercial or financial relationships that could be construed as a potential conflict of interest.

## Supplementary Material

Figure S1 The accumulation of CD4^+^CD28^−^ T-cells in patients with reduced bone mineral density (BMD). Graphs show **(A)** frequencies of freshly isolated CD4^+^CD28^−^ T-cells in patients with normal BMD, osteopenia, and osteoporosis in rheumatoid arthritis (RA) and non-RA cohort; **(B)** frequencies of freshly isolated CD8^+^CD28^−^ T-cells in patients with normal BMD, osteopenia, and osteoporosis in RA and non-RA cohort. **p* ≤ 0.05, **(A,B)** Mann–Whitney *U*-test.

Figure S2 Increased receptor activator of nuclear factor kappa-B ligand (RANKL) expression by CD4^+^CD28^−^ T-cells. Graphs show **(A)** prevalences of RANKL+ cells in freshly isolated naïve CD4^+^CD28^+^CD45RA^+^ T-cells (light green), memory CD4^+^CD28^+^CD45RO^+^ T-cells (dark green), and senescent CD4^+^CD28^−^ T-cells (blue) of rheumatoid arthritis (RA) patients and **(B)** the non-RA cohort; **(C)** median fluorescence intensity (MFI) of intracellular RANKL in freshly isolated naïve CD4^+^CD28^+^CD45RA^+^ T-cells (light green), memory CD4^+^CD28^+^CD45RO^+^ T-cells (dark green), and senescent CD4^+^CD28^−^ T-cells (blue) of RA patients. For all experiments, cells were analyzed directly ex vivo. **p* ≤ 0.05, **(A–C)** Mann–Whitney *U*-test.

Figure S3 Increased bone resorption of CD4^+^CD28^−^ T-cells. Representative microscopic images of bone resorption plates. We cultured 1.5 X 10^5^ monocytes in DMEM with 20 ng/mL M-CSF and 10 ng/mL TGF-β without T cells **(A)**, or 3 X 10^5^ CD^4+^CD28^+^ **(B)**, or 3 X 10^5^ CD^4+^CD28^−^ T-cells **(C)**, in bone resorption assay plates (Cosmo Bio) in the presence of 10 ng/ml sRANKL for 14 days. Monocytes stimulated with 30 ng/ml sRANKL served as a positive control **(D)**. Black indicates areas of resorption.

## References

[1] Sinigaglia L Varenna M Girasole G Bianchi G. Epidemiology of osteoporosis in rheumatic diseases. Rheum Dis Clin North Am (2006) 32:631–58. 10.1016/j.rdc.2006.07.002 17288969

[2] D’Elia HF Larsen A Waltbrand E Kvist G Mellstrom D Saxne T Radiographic joint destruction in postmenopausal rheumatoid arthritis is strongly associated with generalised osteoporosis. Ann Rheum Dis (2003) 62:617–23. 10.1136/ard.62.7.617 12810422 PMC1754591

[3] Theill LE Boyle WJ Penninger JM. RANK-L and RANK: T cells, bone loss, and mammalian evolution. Annu Rev Immunol (2002) 20:795–823. 10.1146/annurev.immunol.20.100301.064753 11861618

[4] Konig A Muhlbauer RC Fleisch H. Tumor necrosis factor alpha and interleukin-1 stimulate bone resorption in vivo as measured by urinary [3H]tetracycline excretion from prelabeled mice. J Bone Miner Res (1988) 3:621–7. 10.1002/jbmr.5650030607 3266953

[5] Kong YY Feige U Sarosi I Bolon B Tafuri A Morony S Activated T cells regulate bone loss and joint destruction in adjuvant arthritis through osteoprotegerin ligand. Nature (1999) 402:304–9. 10.1038/35005552 10580503

[6] Pacifici R. Estrogen deficiency, T cells and bone loss. Cell Immunol (2008) 252:68–80. 10.1016/j.cellimm.2007.06.008 17888417

[7] Mori G D’Amelio P Faccio R Brunetti G. Bone-immune cell crosstalk: bone diseases. J Immunol Res (2015) 2015:108451. 10.1155/2015/108451 26000310 PMC4427089

[8] Mori G D’Amelio P Faccio R Brunetti G. The interplay between the bone and the immune system. Clin Dev Immunol (2013) 2013:720504. 10.1155/2013/720504 23935650 PMC3725924

[9] Goronzy JJ Matteson EL Fulbright JW Warrington KJ Chang-Miller A Hunder GG Prognostic markers of radiographic progression in early rheumatoid arthritis. Arthritis Rheum (2004) 50:43–54. 10.1002/art.11445 14730598

[10] Warrington KJ Takemura S Goronzy JJ Weyand CM. CD4+, CD28- T cells in rheumatoid arthritis patients combine features of the innate and adaptive immune systems. Arthritis Rheum (2001) 44:13–20. 10.1002/1529-0131(200101)44:1<13::AID-ANR3>3.0.CO;2-6 11212151

[11] Koetz K Bryl E Spickschen K O’Fallon WM Goronzy JJ Weyand CM. T cell homeostasis in patients with rheumatoid arthritis. Proc Natl Acad Sci U S A (2000) 97:9203–8. 10.1073/pnas.97.16.9203 10922071 PMC16846

[12] Zhang X Nakajima T Goronzy JJ Weyand CM. Tissue trafficking patterns of effector memory CD4+ T cells in rheumatoid arthritis. Arthritis Rheum (2005) 52:3839–49. 10.1002/art.21482 16329093

[13] Fessler J Raicht A Husic R Ficjan A Duftner C Schwinger W Premature senescence of T-cell subsets in axial spondyloarthritis. Ann Rheum Dis (2016) 75(4):748–54. 10.1136/annrheumdis-2014-206119 25688074 PMC4819616

[14] Solomon DH Karlson EW Rimm EB Cannuscio CC Mandl LA Manson JE Cardiovascular morbidity and mortality in women diagnosed with rheumatoid arthritis. Circulation (2003) 107:1303–7. 10.1161/01.CIR.0000054612.26458.B2 12628952

[15] van der Linden S Valkenburg HA Cats A. Evaluation of diagnostic criteria for ankylosing spondylitis. A proposal for modification of the New York criteria. Arthritis Rheum (1984) 27:361–8. 10.1002/art.1780270401 6231933

[16] Anon. Assessment of fracture risk and its application to screening for postmenopausal osteoporosis. Report of a WHO Study Group. World Health Organ Tech Rep Ser (1994) 843:1–129. 7941614

[17] Kanis JA Johnell O Oden A Johansson H McCloskey E FRAX^TM^ and the assessment of fracture probability in men and women from the UK. Osteoporos Int (2008) 19:385–97. 10.1007/s00198-007-0543-5 18292978 PMC2267485

[18] Aletaha D Smolen J. The Simplified Disease Activity Index (SDAI) and the Clinical Disease Activity Index (CDAI): a review of their usefulness and validity in rheumatoid arthritis. Clin Exp Rheumatol (2005) 23:S100–8. 16273793

[19] Jones DH Kong Y-Y Penninger JM. Role of RANKL and RANK in bone loss and arthritis. Ann Rheum Dis (2002) 61(Suppl 2):ii32–9. 10.1136/ard.61.suppl_2.ii32 12379618 PMC1766717

[20] Djaafar S Pierroz DD Chicheportiche R Zheng XX Ferrari SL Ferrari-Lacraz S. Inhibition of T cell-dependent and RANKL-dependent osteoclastogenic processes associated with high levels of bone mass in interleukin-15 receptor-deficient mice. Arthritis Rheum (2010) 62:3300–10. 10.1002/art.27645 20617528

[21] Zhao B Ivashkiv LB. Negative regulation of osteoclastogenesis and bone resorption by cytokines and transcriptional repressors. Arthritis Res Ther (2011) 13:234. 10.1186/ar3379 21861861 PMC3239342

[22] Braun T Zwerina J. Positive regulators of osteoclastogenesis and bone resorption in rheumatoid arthritis. Arthritis Res Ther (2011) 13:235. 10.1186/ar3380 21861862 PMC3239343

[23] Suzuki Y Mizushima Y. Osteoporosis in rheumatoid arthritis. Osteoporos Int (1997) 7(Suppl 3):S217–22. 10.1007/BF03194376 9536336

[24] Broadley I Pera A Morrow G Davies KA Kern F Expansions of cytotoxic CD4+CD28− T cells drive excess cardiovascular mortality in rheumatoid arthritis and other chronic inflammatory conditions and are triggered by CMV infection. Front Immunol (2017) 8:195 10.3389/fimmu.2017.00195 28303136 PMC5332470

[25] Téo FH de Oliveira RTD Mamoni RL Ferreira MCS Nadruz W Coelho OR Characterization of CD4+CD28null T cells in patients with coronary artery disease and individuals with risk factors for atherosclerosis. Cell Immunol (2013) 281:11–9. 10.1016/j.cellimm.2013.01.007 23416719

[26] Li Y Terauchi M Vikulina T Roser-Page S Weitzmann MN. B cell production of both OPG and RANKL is significantly increased in aged mice. Open Bone J (2014) 6:8–17. 10.2174/1876525401406010008 25984250 PMC4429037

[27] Takeshita S Fumoto T Naoe Y Ikeda K. Age-related marrow adipogenesis is linked to increased expression of RANKL. J Biol Chem (2014) 289:16699–710. 10.1074/jbc.M114.547919 24753250 PMC4059115

[28] Tchkonia T Zhu Y van Deursen J Campisi J Kirkland JL. Cellular senescence and the senescent secretory phenotype: therapeutic opportunities. J Clin Invest (2013) 123:966–72. 10.1172/JCI64098 23454759 PMC3582125

[29] McInnes IB al-Mughales J Field M Leung BP Huang FP Dixon R The role of interleukin-15 in T-cell migration and activation in rheumatoid arthritis. Nat Med (1996) 2:175–82. 10.1038/nm0296-175 8574962

[30] Petrovic-Rackov L Pejnovic N. Clinical significance of IL-18, IL-15, IL-12 and TNF-alpha measurement in rheumatoid arthritis. Clin Rheumatol (2006) 25:448–52. 10.1007/s10067-005-0106-0 16362448

[31] Knevel R Krabben A Brouwer E Posthumus MD Wilson AG Lindqvist E Genetic variants in IL15 associate with progression of joint destruction in rheumatoid arthritis: a multicohort study. Ann Rheum Dis (2012) 71:1651–7. 10.1136/annrheumdis-2011-200724 22440823

[32] Park MK Her Y-M Cho ML Oh H-J Park E-M Kwok S-K IL-15 promotes osteoclastogenesis via the PLD pathway in rheumatoid arthritis. Immunol Lett (2011) 139:42–51. 10.1016/j.imlet.2011.04.013 21620893

[33] Okabe I Kikuchi T Mogi M Takeda H Aino M Kamiya Y IL-15 and RANKL play a synergistically important role in osteoclastogenesis. J Cell Biochem (2017) 118:739–47. 10.1002/jcb.25726 27608420

