# Supplementary figures and images for: Senescent T-Cells Promote Bone Loss in Rheumatoid Arthritis
Source: Front Immunol. 2018 Feb 1;9:95. doi: 10.3389/fimmu.2018.00095 (PMC5810289; doi:10.3389/fimmu.2018.00095)

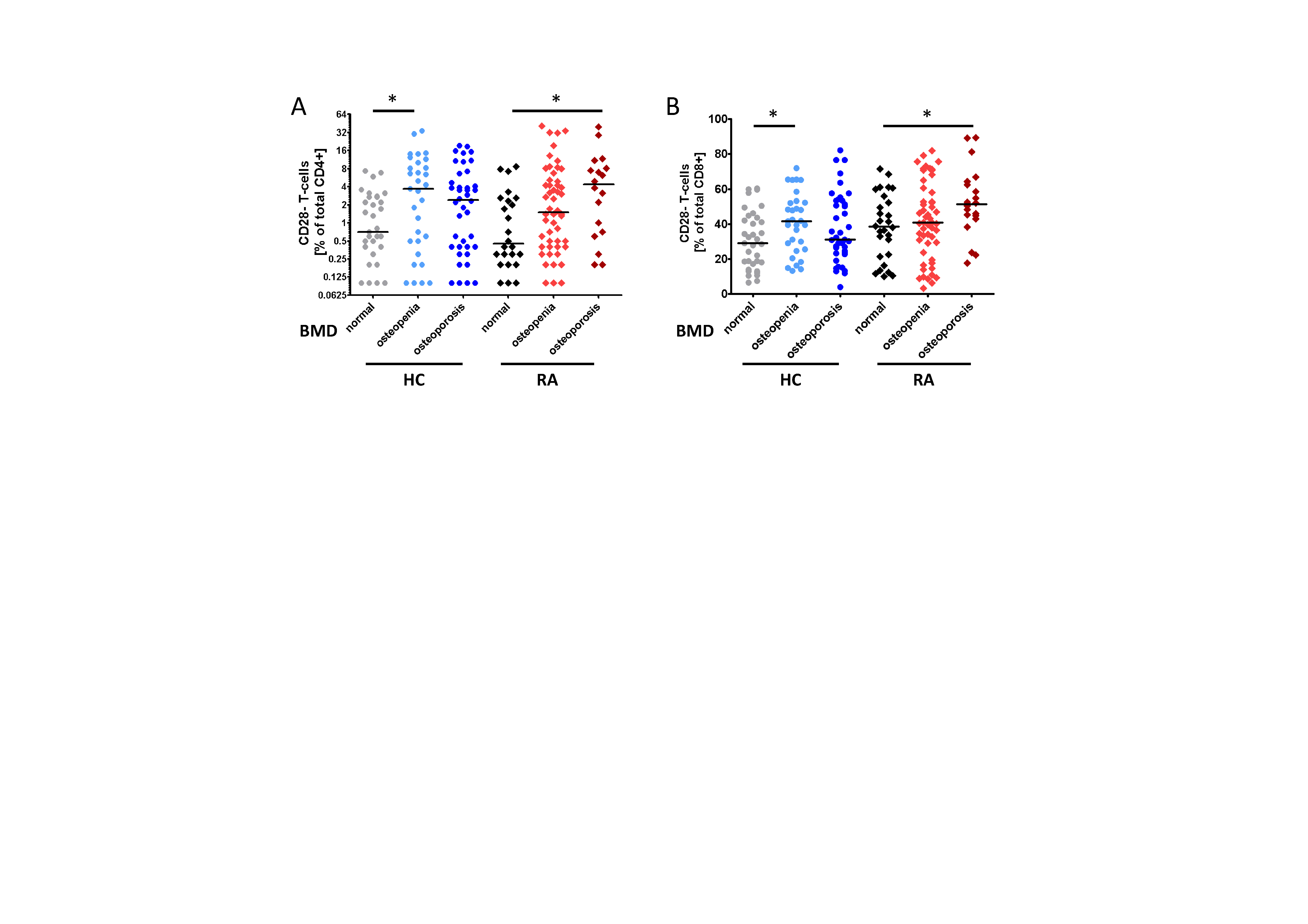

Supplement: Figure S1 — The accumulation of CD4+CD28− T-cells in patients with reduced bone mineral density (BMD). Graphs show (A) frequencies of freshly isolated CD4+CD28− T-cells in patients with normal BMD, osteopenia, and osteoporosis in rheumatoid arthritis (RA) and non-RA cohort; (B) frequencies of freshly isolated CD8+CD28− T-cells in patients with normal BMD, osteopenia, and osteoporosis in RA and non-RA cohort. *p ≤ 0.05, (A,B) Mann–Whitney U-test. [file Image_1.tif]

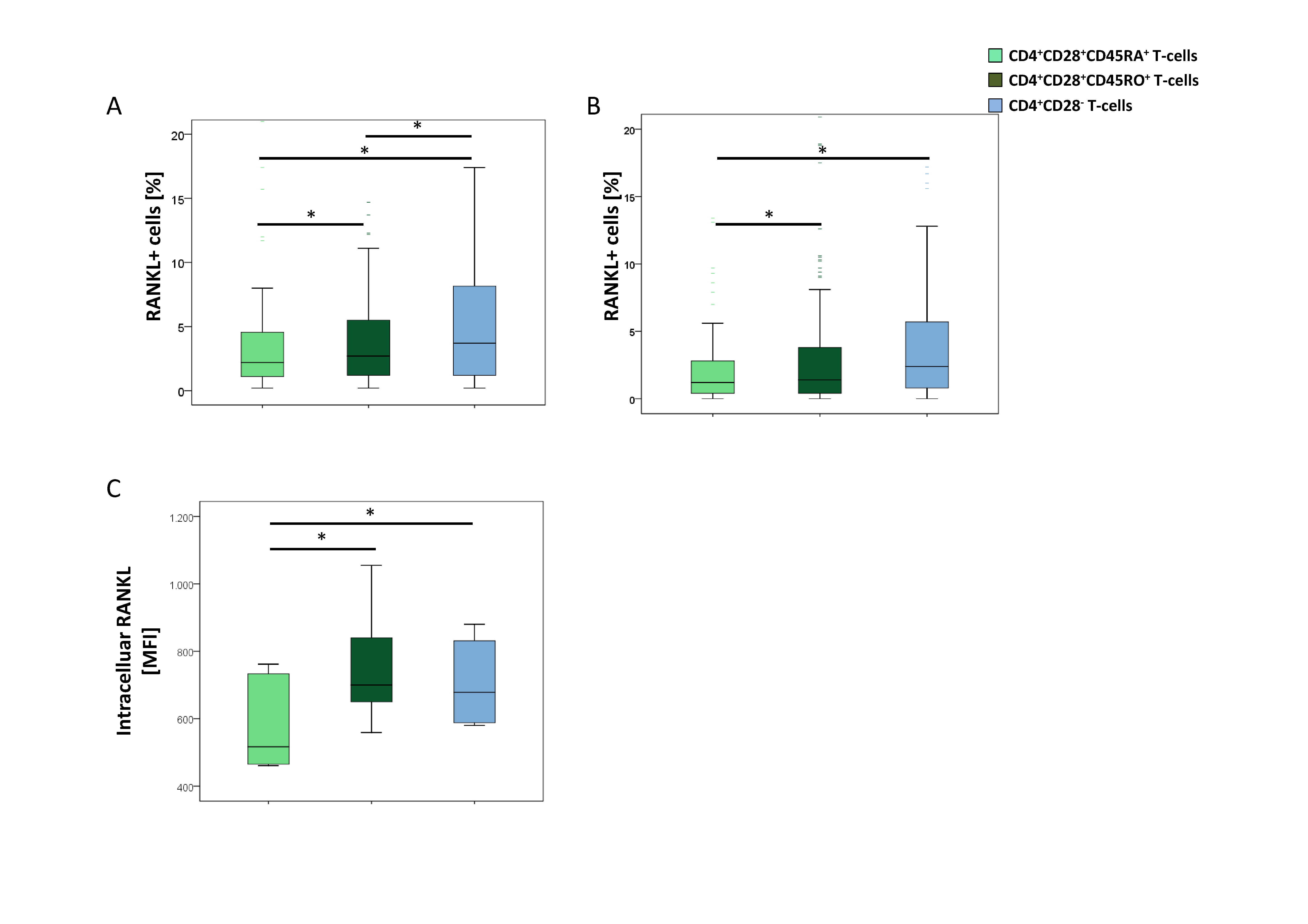

Supplement: Figure S2 — Increased receptor activator of nuclear factor kappa-B ligand (RANKL) expression by CD4+CD28− T-cells. Graphs show (A) prevalences of RANKL+ cells in freshly isolated naïve CD4+CD28+CD45RA+ T-cells (light green), memory CD4+CD28+CD45RO+ T-cells (dark green), and senescent CD4+CD28− T-cells (blue) of rheumatoid arthritis (RA) patients and (B) the non-RA cohort; (C) median fluorescence intensity (MFI) of intracellular RANKL in freshly isolated naïve CD4+CD28+CD45RA+ T-cells (light green), memory CD4+CD28+CD45RO+ T-cells (dark green), and senescent CD4+CD28− T-cells (blue) of RA patients. For all experiments, cells were analyzed directly ex vivo. *p ≤ 0.05, (A–C) Mann–Whitney U-test. [file Image_2.tif]

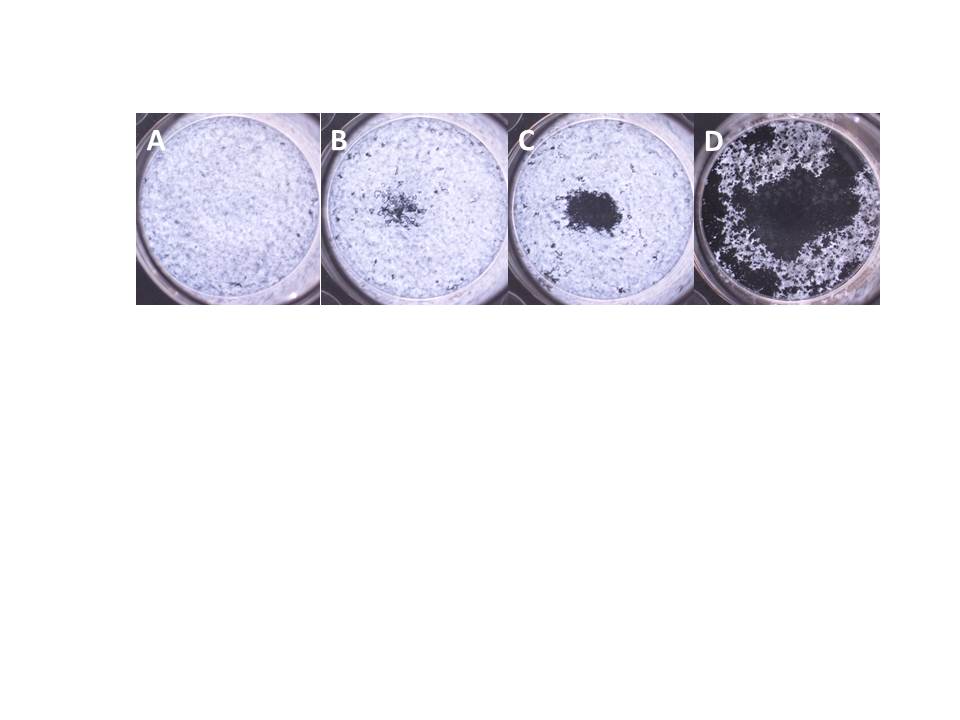

Supplement: Figure S3 — Increased bone resorption of CD4+CD28− T-cells. Representative microscopic images of bone resorption plates. We cultured 1.5 X 105 monocytes in DMEM with 20 ng/mL M-CSF and 10 ng/mL TGF-β without T cells (A), or 3 X 105 CD4+CD28+ (B), or 3 X 105 CD4+CD28− T-cells (C), in bone resorption assay plates (Cosmo Bio) in the presence of 10 ng/ml sRANKL for 14 days. Monocytes stimulated with 30 ng/ml sRANKL served as a positive control (D). Black indicates areas of resorption. [file Image_3.jpeg]
