# Supplementary material for: Senescent T-Cells Promote Bone Loss in Rheumatoid Arthritis
Source: Front Immunol. 2018 Feb 1;9:95. doi: 10.3389/fimmu.2018.00095 (PMC5810289; doi:10.3389/fimmu.2018.00095)
Supplement: Supplementary file 4 [file Table_1.docx]

Suppl. table I: Baseline laboratory parameters

|  | Non-RA | RA | p-value |
| --- | --- | --- | --- |
| Leukocytes [G/l]^‡^ | 5.8 (3.2-12.7) | 6.9 (3.2-15.6) | <0.001 |
| Erythrocytes [T/l]^‡^ | 4.7 (3.7-5.7) | 4.5 (3.7-5.9) | 0.042 |
| Hemoglobin [g/dl]^†^ | 13.8 (±1.1) | 13.5 (±1.3) | 0.101 |
| Hematocrit [%]^†^ | 40.1 (±3) | 40.1 (±3.5) | 0.753 |
| Thrombocytes [G/l]^‡^ | 233 (138-391) | 260.7 (109-450) | 0.113 |
| Creatinine [mg/dl]^‡^ | 0.8 (0.5-1.4) | 0.8 (0.5-1.8) | 0.447 |
| Alanine aminotransferase [U/l]^‡^ | 21 (1-79) | 22 (8-99) | 0.059 |
| B-type natriuretic peptide [pg/ml]^‡^ | n.d. | 110 (5-1779) |  |
| CRP [mg/l]^‡^ | n.d. | 3.3 (0.6-99) |  |
| RF [U/ml]^‡^ | n.d. | 32.5 (1-1219) |  |
| Serum iron [μ/dl]^‡^ | 92 (37-179) | 87.5 (21-216) | 0.315 |
| Transferrin [g/l]^‡^ | 2535.2 (±434.8) | 2574.2 (±398.9) | 0.376 |
| Transferrin saturation [%]^‡^ | 27 (8-58) | 25 (6-59) | 0.264 |
| Ferritin [ng/ml]^‡^ | n.d. | 91 (11-785) |  |
| Soluble transferrin receptor [mg/l]^‡^ | n.d. | 1.35 (0.8-3.28) |  |
| IgG [%]^‡^ | n.d. | 13.2 (6.2-22.3) |  |
| IL-6 [pg/ml]^‡^ | n.d. | 3.9 (1.5-76.3) |  |
| Urine calcium [mmol/l]^‡^ | n.d. | 1.9 (0-15.2) |  |
| Urine calcium creatinine ratio [mmol/mm]^‡^ | n.d. | 0.3 (0-1.6) |  |
| Urine phosphate [mmol/l]^‡^ | 10.1 (2.1-25.7) | 11.2 (0.6-129) | 0.172 |
| CMV (IgG) [U/ml]^‡^ | 119 (0-458) | 140.5 (0-542) | 0.705 |
| CMV (IgM) [U/ml]^‡^ | 0.11 (0.05-1.46) | 0.17 (0.04-4.11) | 0.155 |
| Thyroid-stimulating hormone [μU/ml]^‡^ | 1.2 (0-4.8) | 1.3 (0.1-132) | 0.424 |
| Parathyroid hormone [pg/ml]^‡^ | 44.3 (23.2-104.6) | 42.9 (10.8-108.9) | 0.876 |
| Vitamin D3 [ng/ml]^‡^ | 33.9 (11.7-98) | 29.8 (10-68.1) | 0.009 |
| Bone alkaline phosphatase [μg/l]^‡^ | n.d. | 27.3 (7.3-96.3) |  |
| Osteocalcin [ng/ml]^‡^ | n.d. | 22.9 (6.9-278.1) |  |
| Osteoprotegerin [pmol/l]^‡^ | n.d. | 3.8 (0.7-9.1) |  |
| Soluble RANKL [pmol/l]^‡^ | n.d. | 0.1 (0.1-1.5) |  |
| β-CrossLaps [ng/ml]^‡^ | n.d. | 0.3 (0.1-0.9) |  |

^†^mean (±standard deviation); ^‡^median (range);
