# Supplementary material for: Senescent T-Cells Promote Bone Loss in Rheumatoid Arthritis
Source: Front Immunol. 2018 Feb 1;9:95. doi: 10.3389/fimmu.2018.00095 (PMC5810289; doi:10.3389/fimmu.2018.00095)
Supplement: Supplementary file 5 [file Table_2.docx]

Suppl. Table II: Fracture risk assessment tool (FRAX) data

| FRAX | Non-RA | RA | p-value |
| --- | --- | --- | --- |
| 10 years probability (%)^‡^ | 24.8 (16.6-38.2) | 25.5 (16.6-46.5) | 0.071 |
| Major osteoporotic (%)^‡^ | 6.9 (2.5-63) | 11 (3.5-65) | <0.001 |
| Hip fracture (%)^‡^ | 1.2 (0-53) | 2.3 (0-54) | 0.029 |

^‡^median (range);

The FRAX algorithms give the 10-year probability of fracture. The output is a 10-year probability of hip fracture and the 10-year probability of a major osteoporotic fracture (clinical spine, forearm, hip or shoulder fracture).

Link to FRAX tool: https://www.sheffield.ac.uk/FRAX/tool.jsp?lang=en
