# Supplementary material for: Senescent T-Cells Promote Bone Loss in Rheumatoid Arthritis
Source: Front Immunol. 2018 Feb 1;9:95. doi: 10.3389/fimmu.2018.00095 (PMC5810289; doi:10.3389/fimmu.2018.00095)
Supplement: Supplementary file 6 [file Table_3.docx]

Suppl. table III: Genetic predisposition for lactose intolerance

| LCT (13910) locus* | Non-RA | RA |
| --- | --- | --- |
| CC, n (%) | 16 (24.2) | 22 (21.4) |
| CT, n (%) | 31 (47) | 46 (44.7) |
| TT, n (%) | 19 (28.8) | 35 (34) |

*Lactase gene, chromosome 2; CC reflects for a genetic determined propensity for primary, adult lactose intolerance
