# Supplementary material for: Senescent T-Cells Promote Bone Loss in Rheumatoid Arthritis
Source: Front Immunol. 2018 Feb 1;9:95. doi: 10.3389/fimmu.2018.00095 (PMC5810289; doi:10.3389/fimmu.2018.00095)
Supplement: Supplementary file 7 [file Table_4.docx]

*Supplementary Table IV:* Clinical characteristics of RA patients at follow-up visits:

| months | 0 | 6 | 12 | 18 | 24 |
| --- | --- | --- | --- | --- | --- |
| Number | 107 | 87 | 86 | 77 | 63 |
| Age years]^†^ | 62.6 (±11.5) | 61.4 (±11.2) | 62.2  (±9.7) | 62.1  (±9.7) | 62  (±9) |
| Female, n (%) | 81 (75.7) | 65 (74.7) | 66 (76.7) | 57 (74) | 48 (76.2) |
| Disease duration [years]^‡^ | 12.3 (0-46) | 12.3 (0-46) | 12.3 (0-46) | 12.4 (0-46) | 14 (0-46) |
| Bone mineral density: |  |  |  |  |  |
| Number | 105 | 85 | 84 | 75 | 61 |
| normal, n (%) | 28 (26.7) | 21 (24.7) | 22 (26.2) | 19 (25.3) | 14 (23) |
| osteopenia, n (%) | 55 (52.4) | 47 (55.3) | 49 (58.3) | 46 (61.3) | 39 (63.9) |
| osteoporosis, n (%) | 22 (21) | 17 (20) | 13 (15.5) | 10 (13.3) | 8 (13.1) |
| Disease activity scores: |  |  |  |  |  |
| SDAI^‡^ | 12.1  (0-50.7) | 12.3  (0-47.4) | 11.7  (0-47.4) | 10.8  (0-47.4) | 10.8  (0.1-47.4) |
| DAS28^‡^ | 3.3  (0.3-7.1) | 3.3  (1.1-6.6) | 3.3  (1.1-6.6) | 3.2  (1.1-6.6) | 3.2  (1.1-6.6) |
| Laboratory data: |  |  |  |  |  |
| ESR (mm/1^st^h)^‡^ | 15 (1-66) | 15 (1-66) | 15 (1-66) | 14 (1-66) | 14.5 (1-66) |
| CRP [mg/l]^‡^ | 3.5 (0-52) | 3 (0-52) | 4 (0-52) | 4 (0-52) | 3 (0-52) |
| Current medication: |  |  |  |  |  |
| Corticosteroids, n (%) | 25 (23.4) | 21 (24.1) | 18 (20.9) | 15 (19.5) | 11 (17.5) |
| Biologicals, n (%) |  |  |  |  |  |
| *anti-TNFα* | 27 (25.2) | 23 (26.4) | 24 (27.9) | 22 (28.6) | 17 (27) |
| *Tocilizumab* | 6 (5.6) | 5 (5.7) | 6 (7) | 5 (6.5) | 4 (6.3) |
| *Abatacept* | 13 (12.1) | 11 (12.6) | 11 (12.8) | 9 (11.7) | 10 (15.9) |
| *Rituximab* | 3 (2.8) | 2 (2.3) | 2 (2.3) | 1 (1.3) | 1 (1.6) |
| DMARDs, n (%) |  |  |  |  |  |
| *Methotrexate* | 59 (55.1) | 47 (54) | 47 (54.7) | 43 (55.8) | 34 (54) |
| *Leflunomide* | 16 (15) | 13 (14.9) | 14 (16.3) | 14 (18.2) | 11 (17.5) |
| *Sulfasalazine* | 6 (5.5) | 5 (5.7) | 4 (4.7) | 2 (2.6) | 2 (3.2) |
| *Other* | 5 (4.7) | 4 (4.6) | 3 (3.5) | 2 (2.6) | 3 (4.8) |
| NSAIDs, n (%) |  |  |  |  |  |
| *Regularly* | 13 (12.1) | 12 (13.8) | 10 (11.6) | 9 (11.7) | 9 (14.3) |
| *on demand* | 74 (69.2) | 56 (64.4) | 60 (69.8) | 56 (72.7) | 44 (69.8) |

^†^mean (±standard deviation); ^‡^median (range)

CRP, C-reactive protein [0-5 mg/l]; DAS28, Disease Activity Score 28; DMARD, disease-modifying anti-rheumatic drugs; ESR, erythrocyte sedimentation rate [0-30 mm/h]; n, number; NSAID, non-steroidal anti-inflammatory drugs; RA, rheumatoid arthritis; sDAI, simplified disease activity index
